# Characterization of sequentially-staged cancer cells using electrorotation

**DOI:** 10.1371/journal.pone.0222289

**Published:** 2019-09-19

**Authors:** Claudia I. Trainito, Daniel C. Sweeney, Jaka Čemažar, Eva M. Schmelz, Olivier Français, Bruno Le Pioufle, Rafael V. Davalos

**Affiliations:** 1 CNRS SATIE Institut d’Alembert ENS Paris Saclay, France; 2 Department of Biomedical Engineering and Mechanics, Virginia Tech, Blacksburg, Virginia, United States of America; 3 Department of Human Nutrition, Food, and Exercise, Virginia Tech, Blacksburg, Virginia, United States of America; 4 ESIEE-Paris, ESYCOM (FRE 2028), UPE, Noisy-Le-Grand, France; Texas A&M University College Station, UNITED STATES

## Abstract

The identification and separation of cells from heterogeneous populations is critical to the diagnosis of diseases. Label-free methodologies in particular have been developed to manipulate individual cells using properties such as density and morphology. The electrical properties of malignant cells, including the membrane capacitance and cytoplasmic conductivity, have been demonstrated to be altered compared to non-malignant cells of similar origin. Here, we exploit these changes to characterize individual cells in a sequentially-staged *in vitro* cancer model using electrorotation (EROT)—the rotation of a cell induced by a rotating electric field. Using a microfabricated device, a dielectrophoretic force to suspend cells while measuring their angular velocity resulting from an EROT force applied at frequencies between 3 kHz to 10 MHz. We experimentally determine the EROT response for cells at three stages of malignancy and analyze the resultant spectra by considering models that include the effect of the cell membrane alone (single-shell model) and the combined effect of the cell membrane and nucleus (double-shell model). We find that the cell membrane is largely responsible for a given cell’s EROT response between 3 kHz and 10 MHz. Our results also indicate that membrane capacitance, membrane conductance, and cytoplasmic conductivity increase with an increasingly malignant phenotype. Our results demonstrate the potential of using electrorotation as a means making of non-invasive measurements to characterize the dielectric properties of cancer cells.

## Introduction

The processes of identification, selection, and separation of cells from complex, heterogeneous sample populations are of fundamental importance in the development of novel cancer diagnostic tests and treatments. Cancer presents in a number of different forms, which affect various tissues and have different characteristics depending on the origin tissue and degree of malignancy. However, tumors typically appear with several common characteristics, including the capacity for self-proliferation and aggressiveness towards the host’s other cells and tissues [1]. Cancer treatments seek to abate tumor growth and proliferation, though many of these techniques, such as resection and chemotherapy, have become known for their brutality. Diagnosing cancerous tissue at earlier stages of pathogenesis could increase patient life expectancy and decrease mortality by enabling treatments to be administered while the tumor is still small and unobstructive. Unfortunately, early cancer detection is often difficult because physical symptoms may be absent during the early stages of tumorogenesis. Nevertheless, the early detection of cancer could mitigate health complications associated with late-stage treatments and enhance overall patient survival rates.

In modern medicine, tumor biomarker analysis plays a central role in cancer diagnosis and evaluation of the risks associated with various cancer therapies. Integration of biomarker technology into the diagnostic and therapeutic process has created a popular research field [2]. For example, the simultaneous analysis of four biomarkers (leptin, prolactin, osteopontin, and insulin-like growth factor-II) within a blood sample can improve the accuracy of early diagnoses of ovarian epithelial cancer to an efficiency of 95% [3]. Furthermore several gene products, detected through unique nucleic acid identifiers and quantified by real-time polymerase chain reaction, have been proposed as biomarkers for the detection of early-stage cancer [4]. However, these processes are time consuming and often require highly-specific equipment or training to perform the relevant tests, and may only be practically implemented in a well-equipped laboratory or clinic, which limits their portability.

Cancer cells exhibit different physical properties compared to normal cells; several of which have been investigated for use in diagnosing cancer. Biomarker-independent methods have been developed *in vitro* in order to distinguish malignant cells from normal cells based on intrinsic properties, such as volume [5], mechanical deformation [6, 7], and response to an electric field [8–11]. It has been demonstrated that normal and malignant cells show significant differences in proliferation and metabolic mechanisms, cytoskeletal structure, and in other phenotpyes [12]. For instance, the membrane capacitance, which reflects the morphological changes occurring on the cell surface, is commonly altered during cellular pathogenesis. For example, Leukemia and other cancer cells have decreased membrane capacitance than normal T lymphocytes and erythrocytes [13, 14]. Other parameters, such as electrical impedance, have been used to differentiate breast cancer cells from those in the surrounding tissues [15]. Understanding the manipulations that occur during the stages of cancer could provide an avenue for better understanding biophysical changes associated with cancer and malignant cell phenotypes that could serve as the basis for future early screening technologies.

To this end, the identification and study of the dielectric properties of cancer cells through their response to applied electric fields could provide a promising means characterize early-stage cancer cells. Electrokinetic phenomena such as dielectrophoresis, traveling wave dielectrophoresis, and electrorotation (EROT) have provided particularly interesting means of cellular manipulation and have been integrated into lab-on-chip platforms [16, 17]. These methods are based on the electrical polarizability of cells and consist of applying a non-uniform AC electric field to the cell. Dielectrophoresis is the phenomenon in which local electric field gradients create a differential charge density within a cell. This differential polarization results in an electrically-driven translation in the direction of the local electric field gradient. A cell’s electrical polarization has a dependence on the frequency of the applied electric field, the volume of the cell, and the dielectric characteristics of both the cell and the external medium [18–20]. DEP has been used to identify electrical properties that differ between normal and cancerous cells [11, 21, 22]. Recent innovations in DEP-based technologies have enabled the sterility of the sample to be maintained while mitigating electrochemical effects at fluid-electrode boundaries [9].

If immersed within multiple AC electric fields that are out of phase, the mechanical forces applied to a cell will induce a rotational velocity. Electrorotation (EROT) describes the purely-rotational motion of a cell within a phased electric field. In EROT-based devices designed to manipulate cells, electrodes are often arranged in circle with a phase delays between the electric fields applied between consecutive electrodes. The net effect of this phased array is the creation of a rotational electric field with cells contained within it experiencing a torque [23]. As in DEP, the rotational velocity of the cell experiencing EROT depends on the dielectric properties of both the cell itself and its surrounding medium, in addition to the frequency and strength of the applied electrical field [23–25].

The differences in a cell’s rotational velocity at different frequencies enable the estimation of its electrophysiological properties from its characteristic EROT spectrum. These frequency-dependent responses create electrical signatures that can be used to distinguish malignant cells from healthy ones [14, 24–26]. These differences are intrinsic to each pathological stage and depend on cellular structure and physiology, such as microvilli, membrane folding, and protrusions [11, 21, 27, 28]. For example, it has been demonstrated that the increasing invasiveness correlates to increased membrane ruffling, which increases membrane capacitance, in cancer cells [29]. Membrane protrusions present in more aggressive cellular phenotypes result in greater membrane conductivity. Additionally, more invasive cancer cells exhibit an altered cytoskeleton morphology, which affects their morphology and viscoelasticity, which also impact a cell’s electrical signature [12].

Here, we describe the development and implementation of a method to use the combination of DEP and EROT to elucidate the phenotypic differences between sequentially staged mouse ovarian surface epithelial cancer cells. We demonstrate differences between early-stage, late-stage, and highly-aggressive cells, especially at the lower-frequencies studied (between 3 kHz and 100 kHz). We analyze the resultant spectra by considering models that include the effect of the cell membrane alone (single-shell model) and the combined effect of the cell membrane and nucleus (double-shell model). Finally, we demonstrate that cell membrane capacitance and conductance, and cytoplasmic conductivity, increase with increasing phenotypic malignancy. Our method describes the first attempt to identify sequentially-staged cancer cells through their dielectric properties using the combination of DEP and EROT forces. Furthermore, it is complementary to the clinical methods currently in use and may improve the detection accuracy of early-stage cancer diagnostic techniques through its label-free nature and single-cell resolution.

## Materials and methods

### Theoretical framework

Broadly, the use of electric fields to manipulate biological specimens is a promising characterization tool for cell separation and identification. In the presence of a time-harmonic applied electric field, cells become electrically polarized, resulting in an effective charge dipole within each cell [18, 30]. The electrical response of a cell is composed of a translational component (DEP) and a rotational component (EROT). It has been experimentally demonstrated that the field-induced dipole moment of the cell is linked to the applied electric field, which stands in good agreement with the existing theoretical framework [31]. Considering the induced dipole within the cell to be a relatively-small, compared to the inhomogeneities within the electric field, the time-averaged torque applied to the cell 〈**T**_*E*_〉 is given by

$$\begin{array}{c} \left\langle T_{E}\right\rangle =-4\pi \epsilon_{m}R^{3}I\left(K_{CM}\right){\left|E_{rms}\right|}^{2}\hat{z}, \end{array}$$ (1)

where *ϵ*_*m*_ is the permittivity of the medium in which the cell is immersed, *R* is the radius of the cell, $I(K_{CM})$ denotes the imaginary part of the Clausius–Mossotti factor (*K*_*CM*_), and **E**_*rms*_ is the root mean square of the applied electric field. The drag torque 〈**T**_*E*_〉 opposes the electrorotational torque and is given by

$$\begin{array}{c} \left\langle T_{D}\right\rangle =8\pi \eta R^{3}u\hat{z}, \end{array}$$ (2)

where *η* is the dynamic viscosity of the medium and *u* is the angular velocity of the cell. At steady state, 〈**T**_*E*_〉 + 〈**T**_*D*_〉 = 0, yielding an expression for the angular velocity as a function of *ω*,

$$\begin{array}{c} u=-\frac{\varepsilon_{m}}{2\eta }I\left(K_{CM}\right){\left|E_{rms}\right|}^{2}. \end{array}$$ (3)

DEP and EROT have previously been combined in order to assess electrophysiological differences between viable and non-viable yeast [32]. In our work, we apply the same principles (EROT associated with DEP) to demonstrate how the practical combination DEP and EROT may be used identify malignant and benign cells, which could help improve cancer diagnosis and provide insight into appropriate treatment options. Both DEP and EROT depend the frequency-dependent properties of a cell immersed in an electric field which depends on the electrical conductivity and permittivity of the cell and the external medium. The complex permittivity given by

$$\begin{array}{c} \varepsilon_{i}^{*}:=\varepsilon_{i}\varepsilon_{0}-j\frac{\sigma_{i}}{\omega } \end{array}$$ (4)

where *ε*_*i*_ and *σ*_*i*_ are the permittivity and conductivity of a spatial domain, *ε*_0_ is the permittivity of the vacuum, *ω* is the angular frequency of the applied electric field such that *ω* = 2*πf* where *f* is the frequency of the applied signal given in Hertz, and $j=\sqrt{-1}$.

The Clausius-Mossotti factor expresses the dielectric constant of a particle in terms of its polarizability [33, 34] and is defined as

$$\begin{array}{c} K_{CM}=\frac{\varepsilon_{p}^{*}-\varepsilon_{m}^{*}}{\varepsilon_{p}^{*}+2\varepsilon_{m}^{*}}, \end{array}$$ (5)

where $\varepsilon_{p}^{*}$ and $\varepsilon_{m}^{*}$ are the complex permittivities of the cell and the external medium, respectively. *K*_*CM*_ can be both positive and negative, depending on the frequency of the angular applied electric field *ω*. In order to represent $\varepsilon_{p}^{*}$ for a structure such as a cell, a model comprising concentric spherical shells can be developed. Representing a cell as an inner cytoplasmic domain with radius *R* − *t* separated from the external domain by a membrane domain of thickness *t* such that *t* < < *R* is referred to as a single-shell model. For a single-shell model, $\varepsilon_{p}^{*}$ is given by

$$\begin{array}{c} \varepsilon_{p}^{*}=\varepsilon_{mb}^{*}\{\frac{v_{0}+2(\frac{\varepsilon_{cp}^{*}-\varepsilon_{mb}^{*}}{\varepsilon_{cp}^{*}+2\varepsilon_{mb}^{*}})}{v_{0}-(\frac{\varepsilon_{cp}^{*}-\varepsilon_{mb}^{*}}{\varepsilon_{cp}^{*}+2\varepsilon_{mb}^{*}})}\} \end{array}$$ (6)

where $\varepsilon_{cp}^{*}$ is the complex permittivity of the cell interior and $\varepsilon_{mb}^{*}$ is the complex permittivity of the cell membrane, and *v*_0_ = (*R*/(*R* − *t*))^3^.

In the more complex double-shell model, a cell is modeled as two conductive domains with associated radii *R*_*n*_ − *t*_*n*_ and *R* − *t* for the nucleus and the cytoplasm, respectively [20]. The complex permittivities of the nuclear membrane and nucleoplasm are $\varepsilon_{nb}^{*}$ and $\varepsilon_{np}^{*}$. The nuclear domain is separated from the cytoplasmic domain by the nuclear membrane domain with thickness *t*_*n*_ and the cytoplasmic domain is separated from the extracellular domain by a cell membrane with thickness *t*. Explicitly, the double shell model is given by

$$\begin{array}{c} \varepsilon_{p}^{*}=\varepsilon_{mb}^{*}\frac{2\left(1-v_{1}\right)+\left(1+2v_{1}\right)E_{1}}{\left(2+v_{1}\right)+\left(1-v_{1}\right)E_{1}}, \end{array}$$ (7)

where *v*_1_ = (1 − *t*/*R*)^3^ and *E*_1_ is given by

$$\begin{array}{c} E_{1}=\frac{\varepsilon_{cp}^{*}}{\varepsilon_{mb}^{*}}\frac{2\left(1-v_{2}\right)+\left(1+2v_{2}\right)E_{2}}{\left(2+v_{2}\right)+\left(1-v_{2}\right)E_{2}}, \end{array}$$ (8)

where *v*_2_ = (*R*_*n*_/(*R* − *t*))^3^ and *E*_2_ is given by

$$\begin{array}{c} E_{2}=\frac{\varepsilon_{np}^{*}}{\varepsilon_{nb}^{*}}\frac{2\left(1-v_{3}\right)+\left(1+2v_{3}\right)E_{3}}{\left(2+v_{3}\right)+\left(1-v_{3}\right)E_{3}}, \end{array}$$ (9)

where *v*_3_ = (1 − *t*_*n*_/*R*_*n*_)^3^ and $E_{3}=\varepsilon_{np}^{*}/\varepsilon_{nb}^{*}$.

### Cell culture

Cells in the mouse ovarian surface epithelial cell line (MOSE) mimic the progression of ovarian cancer from an early, benign stage (MOSE-E), to a malignant stage (MOSE-L, slow-developing disease) [35], and finally to a late, highly aggressive/invasive stage (MOSE-L_TIC*ν*_, fast-developing disease) [36]. Cells from each of these stages exhibit different phenotypes such as morphology, cytoskeletal architecture, size, metabolism, and growth rates [12, 37]. The three cell lines were routinely cultured in DMEM, supplemented with 4% fetal bovine serum and 1% penicillin/streptomycin. Cells were collected by trypsinization and subsequent centrifugation, washed three times, then resuspended in a low-conductivity (*σ*_*m*_ = 0.01 S/m), isotonic medium consisting of 8.5% sucrose [w/v], 0.3% glucose [w/v], 0.725% RPMI (Life Technologies) 0.1% BSA, [w/v], 0.1% Kolliphor (both from Sigma-Aldrich) and 0.1% EDTA (Boston Bio Products, Ashland, MA) to mitigate the Joule heating and thermal damage that could potentially be generated during the applications of electric fields [10]. Samples of cells from each sub-population—*n* = 14 for the MOSE-E, *n* = 15 for the MOSE-L, and *n* = 13 for the MOSE-L_TIC*ν*_—were then introduced into the device to measure their EROT spectra.

### Device Set-Up

**Fig 1:**
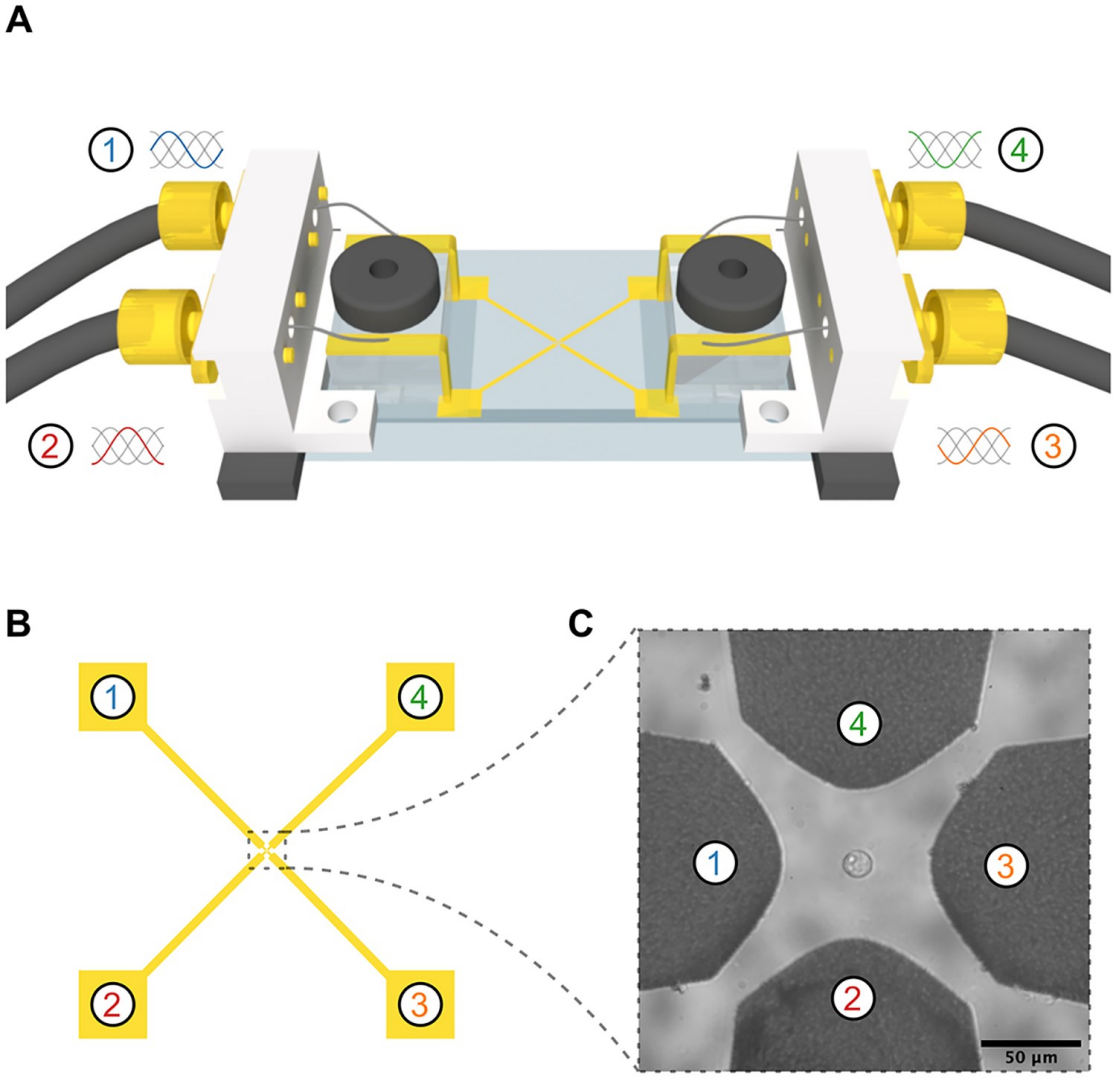
Schematic of the EROT chip. A: A sinusoidal waveform with phases offset 90° were applied to each of four electrodes. A lower-frequency DEP signal was applied to the electrodes to trap the cell during EROT experiments. B: The electrodes were patterned on a glass substrate and C: a single cell was exposed to the 90° offset waveform to obtain its EROT spectrum.

**Fig 2:**
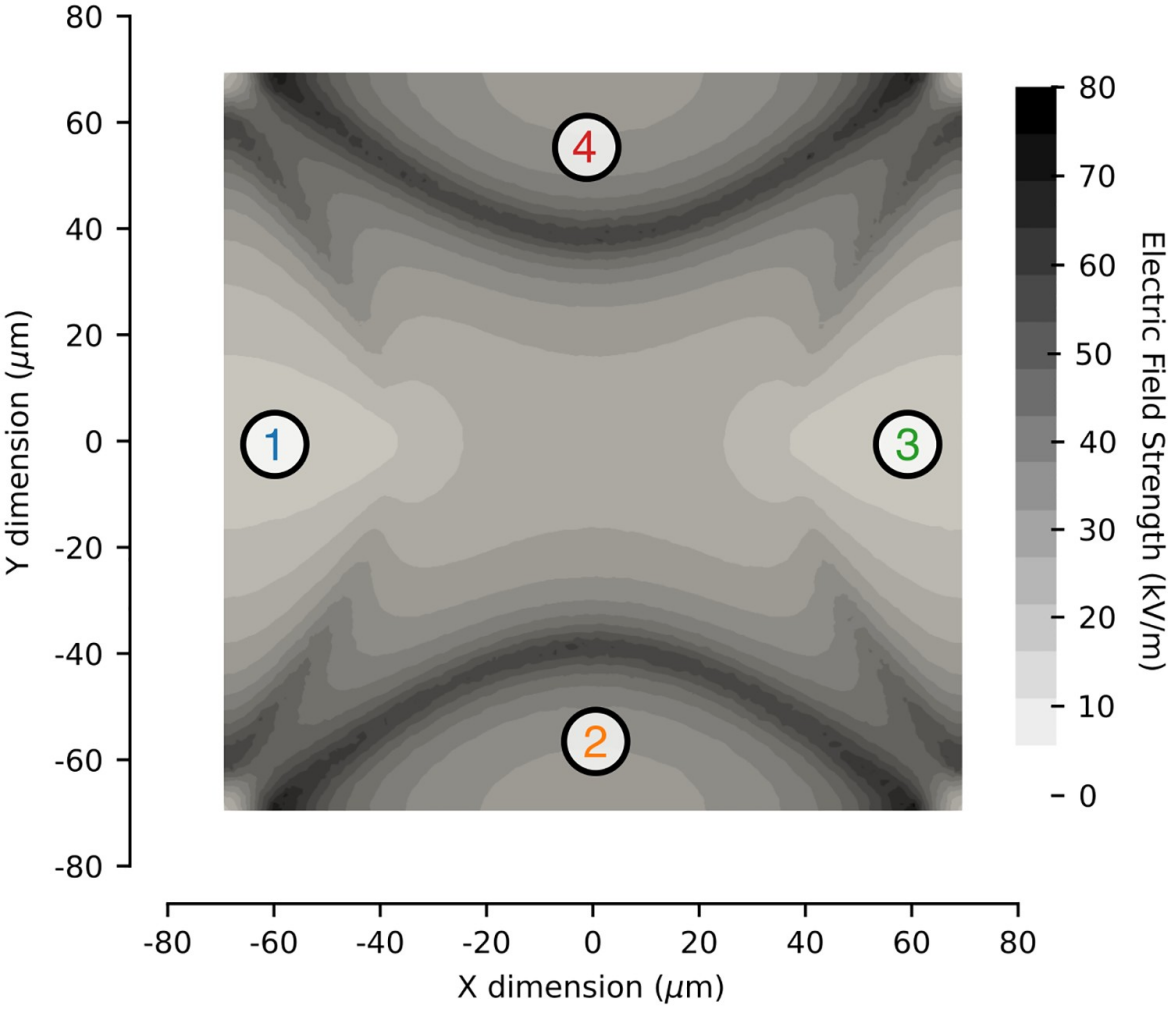
The electric field between the electrodes during the application of the combined DEP-EROT waveform balanced the gravitational force to levitate cells within the device and rotate them around a central vertical axis. A DEP signal was generated at 4 V peak-to-peak voltage sine wave at 10 kHz. Two 90° phase shifted signals were generated by a second function generator at 2 V peak-to-peak and frequencies between 3 kHz and 10 MHz, creating an electric field intensity of 14 kV/m. Frequencies were summed before being applied to the electrodes 1-4, with 90° phase offsets between each adjacent pair of electrodes.

EROT measurements were performed in a custom-built chamber mounted on the microscope setup previously described (Fig 1) [38]. Briefly, the chamber consisted of a standard microscope slide with quadrupolar gold electrodes patterned on the surface (Fig 1A and 1B) [33]. The spacing between electrodes was 75 *μ*m and the cells were positioned within the center of the electrodes (Fig 1C). The electrodes were energized with a time-harmonic electric potential that allowed translational manipulation of cells using DEP and rotational manipulation using a phased EROT signal. The DEP signal was generated by a U2761A function generator (Agilent, Santa Rosa, CA, USA) at 4 V peak-to-peak voltage sine wave at 10 kHz. Two 90° phase shifted signals were generated by a second function generator (Analog Discovery; Digilent, Pullman, WA, USA) at 2 V peak-to-peak and frequencies between 3 kHz and 10 MHz. The electric field intensity was calculated to be 14 kV/m within the center of the electrodes at the position of the cells (Fig 2). The signals were summed using a custom built amplifier.

### Computational methods

EROT videos were processed using a macro written in ImageJ (NIH, Bethesda, MD, USA). The background was subtracted using a median filter, each frame was converted to binary image and an ellipse was fitted to a cell. The change of the ellipse axis angle was exported to MATLAB (Mathworks, Natick, MA, USA) and filtered using moving average filter to remove the noise. The rotational velocity of a cell was calculated as an average change of the angle between each frame in a 15 s video sequence.

Eq 3 was used to fit the single- and double-shell models to the electrotorational velocity spectrum. The radius of each cell, the frequency of the rotational electric field, and the conductivity of the extracellular medium were measured, reducing the number of unknown parameters. Furthermore, the permittivity of the aqueous extracellular medium is well known in literature and was also fixed. Curve fitting was performed using the Levenberg-Marquardt algorithm through the curve_fit() function in the Scipy (v1.1.0) module in Python 3.7 to minimize the sum of square residuals (Eq 10) between the experimental data and Eq 3. A Markov Chain Monte-Carlo (MCMC) algorithm was also used to fit the models to the EROT spectra for each cell type, enabling the goodness of fit to be explored by varying the parameter spaces for each model. Briefly, a sample of a bounded parameter space is used to fit the model, which was then used to evaluate the objective function, Eq 10. Based on this evaluation, an additional sample will be used to re-evaluate the model and Eq 10. This process is repeated, with each iteration depending only on the results of the previous iteration and will generate a series of samples from the parameter space that enable the convergence of the objective function and create probability distributions for each parameters. The emcee module [39] was used to generate parameter estimates using an Affine Invariant Markov Chain Monte-Carlo Ensemble sampler with 20 walkers at 5 temperatures, each taking 10000 steps. No burn-in was used during data analysis due to the large number of steps and multiple temperatures ensuring relatively rapid convergence. The root mean square intensity of the EROT-inducing electric field was calculated using a three-dimensional simulation created using the finite element environment FEniCS (v2018.1.0) [40]. Gmsh (v3.0.1) was used for spatial discretization into linear triangular elements [41].

#### Statistical methods

Following parameter estimation, statistical differences between each cell type were characterized using a one-way analysis of variance. If significance was found, statistical differences between the parameters for each group were compared using a an unpaired t-test.

### Results

#### Parameter estimation

Eq 3 was fit to the experimental EROT spectra for each cell by minimizing the L^2^-norm of the difference between the experimental data and the model

$$\begin{array}{c} SSR=\left|\right|\left\langle u_{ex}\right\rangle -\left\langle u\right\rangle {\left|\right|}_{2}, \end{array}$$ (10)

where *u*_*ex*_ is the experimental data, *u* is given by Eq 3 using the single- and double-shell model. The cells within the device were levitated at approximately 3 *μ*m above the center of the electrodes where the electric field is relatively homogeneous [38]. This height varied for difference cells, but the electric field amplitude remains relatively constant within the center of the electrodes, ranging from 14 kV/m at 0-10 *μ*m above the base between the electrodes to 10 kV/m at 40-50 *μ*m. The applied electric field between the electrodes used in this analysis was calculated to be *E*_*rms*_ = 14 kV/m (Fig 2). During the fitting process, the frequency of the applied electric field *ω* and the cell radius *R* were measured and the remainder of the parameters were fixed at the values in Table 1, except for *σ*_*o*_, *ε*_*m*_, and *R*. The rotational velocity depends on the frequency of the applied rotating electric field and on the complex dielectric properties each of the three cell lines. The cell radius for each cell was measured during experimentation. The ratio of the nuclear radius to the cell radius was calculated based on previously-reported nucleus-to-cytoplasm and cell radius measurements [9]: 0.67 for the Early, 0.59 for the Late, and 0.68 for the MOSE-L_TIC*ν*_. In addition to defining the nuclear and cell radii, the electrical conductivity of the external medium was measured to be 0.01 S/m and with a dielectric constant of 80 [10]. By fixing the known parameters, the single-shell model was parameterized using 5 unknown parameters and the double-shell model using 10 unknown parameters. To confirm our parameter fits, we employed an MCMC algorithm to sample the parameter space (10^6^ total samples) for the single and double shell models. For the MOSE-E, MOSE-L, and MOSE-L_TIC*ν*_ cells, the single- and double-shell models, the AIC and reduced *χ*-squared values are given Table 2, for both fitting methods.

**Table 1:** Physical constants used in calculating EROT spectrum.

Cell Type	Parameter	LM	MCMC	Range	Unit	Description	Reference
All	*ε*_0_	-	-	8.85 × 10^−12^	F/m	Vacuum permittivity	
	*ε*_*m*_	-	-	80		Medium relative permittivity	[42]
	*σ*_*m*_	-	-	0.01	S/m	Medium conductivity	measured
	*η*	-	-	0.89	mPa ⋅ s	Medium dynamic viscosity	[43]
	*E*_*rms*_	-	-	14	kV/m	Electric field magnitude (RMS)	calculated
MOSE-E	*ε*_*mb*_	30	29	[1.0, 30]		Membrane relative permittivity	[7, 27, 44, 45]
	*σ*_*mb*_	32	33	[10^−6^, 10^3^]	*μ*S/m	Membrane conductivity	[7, 27, 44, 46]
	*ε*_*cp*_	45	68	[45, 125]		Cytoplasm relative permittivity	[45]
	*σ*_*cp*_	0.90	0.89	[0.01, 2.0]	S/m	Cytoplasm conductivity	[47]
	*t*	4.0	4.1	[4.0, 40]	nm	Membrane thickness	[7, 48]
MOSE-L	*ε*_*mb*_	30	30	[1.0, 30]		Membrane relative permittivity	[7, 27, 44, 45]
	*σ*_*mb*_	52	52	[10^−6^, 10^3^]	*μ*S/m	Membrane conductivity	[7, 27, 44, 46]
	*ε*_*cp*_	45	80	[45, 125]		Cytoplasm relative permittivity	[45]
	*σ*_*cp*_	1.0	1.0	[0.01, 2.0]	S/m	Cytoplasm conductivity	[47]
	*t*	4.0	4.0	[4.0, 40]	nm	Membrane thickness	[7, 48]
MOSE-L_TIC*ν*_	*ε*_*mb*_	30	30	[1.0, 30]		Membrane relative permittivity	[7, 27, 44, 45]
	*σ*_*mb*_	61	60	[10^−6^, 10^3^]	*μ*S/m	Membrane conductivity	[7, 27, 44, 46]
	*ε*_*cp*_	45	91	[45, 125]		Cytoplasm relative permittivity	[45]
	*σ*_*cp*_	1.3	1.2	[0.01, 2.0]	S/m	Cytoplasm conductivity	[47]
	*t*	4.0	4.0	[4.0, 40]	nm	Membrane thickness	[7, 48]

LM indicates the Levenberg-Marquardt algorithm and MCMC indicates the Markov Chain Monte-Carlo algorithm. For each parameter, the range used in the parameter fits is based on the references cited The parameter values given were obtained by considering the data in aggregate for each cell type during the fitting process.

**Table 2:** Quality of fit metrics for the single- and double-shell models applied to the each the MOSE-E, MOSE-L, and MOSE-L_TIC*ν*_ cells.

Cell Type	Shells	LM AIC	LM *χ*^2^	MCMC AIC	MCMC *χ*^2^
MOSE-E	1	369	10.0	369	10.0
	2	378	10.3	430	14.2
MOSE-L	1	179	2.79	181	2.81
	2	190	2.88	668	48.1
MOSE-L_TIC*ν*_	1	128	2.60	133	2.69
	2	139	2.72	528	55.5

The AIC and reduced *χ*^2^ values are evaluated to assess the quality of fit for each model. LM indicates the Leavenburg-Marquardt algorithm and MCMC indicates the Markov Chain Monte-Carlo algorithm. For the each measure, lower values correspond to better model qualities and represents a lesser degree of information loss for the AIC values and a better data fit for the reduced *χ*^2^ values.

#### Model quality

**Fig 3:**
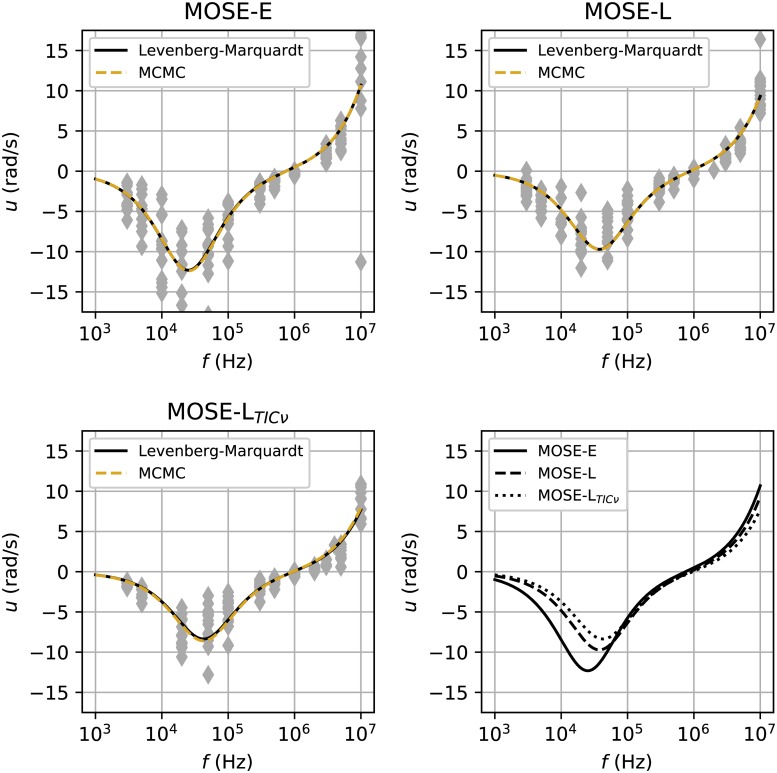
Electrorotational spectra for MOSE cells in order of increasing malignancy: A: MOSE-E, B: MOSE-L, and C: MOSE-L_TIC*ν*_. The solid black line and the gold dashed line in each panel correspond to the best-fit curves generated by the Levenberg-Marquardt and MCMC algorithms, respectively. D: The best-fit curves are superimposed to emphasize the difference in rotational velocity between the three cell types between 3 × 10^3^ and 10^5^ Hz. The parameters used to generate the best-fit curves were generated using both Levenberg-Marquardt and MCMC algorithms and are in good agreement for each cell type. Note that the MCMC and Levenberg-Marquardt parameter estimation methods provide similar parameter estimates and therefore overlapping best-fit curves.

The abilities of the single-shell and double-shell models to describe the rotational spectra for each of the MOSE-E, MOSE-L and MOSE-L_TIC*ν*_ cells were evaluated using the Akaike information criterion (AIC). To compare the relative quality the single- and double-shell models in describing the experimental data, the AIC was calculated for each combination of model and cell type according to the formula

$$\begin{array}{c} AIC_{i,c}=2k_{i}-n_{c}\log\left(SSR\right), \end{array}$$ (11)

where *i* ∈ {*s*, *d*} for the single-shell and double-shell models, respectively. The experimental data was sampled between 3 kHz and 10 MHz for the MOSE-E cells (*n* = 14 cells), the MOSE-L (*n* = 15 cells) and MOSE-L_TIC*ν*_ (*n* = 13). As part of the fitting algorithm, the sum of square residuals was calculated using Eq 10, enabling the relative probability of minimizing information loss given by exp(min{*AIC*_*i*,*s*_ − *AIC*_*i*,*d*_}/2) for each cell type for both the single-shell and double-shell models. In each case, for the MOSE-E, MOSE-L, and MOSE-L_TIC*ν*_ cells the double-shell model had a relative probability of minimizing the information loss of < 0.01%. The single-shell model was therefore selected over the double-shell model for the present study (Fig 3).

#### Electric phenotype changes with malignancy

**Fig 4:**
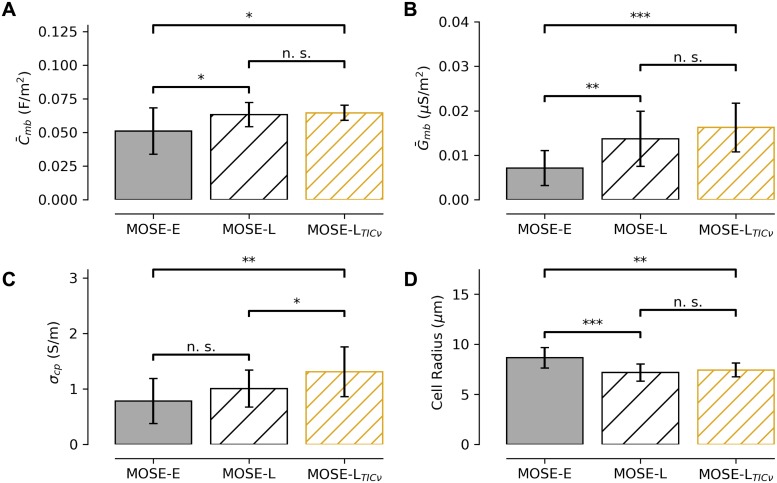
Electrophysiological changes in cellular physiology occur with increasing malignancy. A: The specific capacitance of the cell membrane (${\bar{C}}_{mb}=\varepsilon_{m}\varepsilon_{0}/t$) increases with malignancy in the MOSE cell line, from the MOSE-E (*n* = 14), MOSE-L (*n* = 15), and MOSE-L_TIC*ν*_ (*n* = 13). B: The specific conductance of the cell membrane (${\bar{G}}_{mb}=\sigma_{mb}/t$) and C: the cytoplasmic conductivity (*σ*_*cp*_) also increase with increasingly malignancy. D: The cell radius (*R*) decreases with increasing malignancy between the MOSE-E and MOSE-L cells, but shows little difference between the MOSE-L and MOSE-L_TIC*ν*_ cells. Data are shown as mean ± standard deviation. * indicates *p* < 0.01, ** indicates *p* < 0.001, *** indicates *p* < 0.0001, and n. s. indicates no significant difference.

The MOSE cell line, which includes several sub-lines, is a convenient *in vitro* model of cancer cell progression to increasingly malignant phenotypes. Our data confirms that concurrent changes in specific cell membrane capacitance is associated with this increase in malignancy and cellular invasiveness. For the MOSE-E, MOSE-L, and MOSE-L_TIC*ν*_, the specific cell membrane capacitance (${\bar{C}}_{mb}=\varepsilon_{mb}\epsilon_{0}/t$) was calculated based on our fitting of the rotational spectra from each individual cell (Fig 4A). Our results demonstrate that both a decrease in the cell membrane thickness and an increase in the membrane dielectric constant contribute to this increasing capacitance from the MOSE-E cells, to the MOSE-L cells, but no statistical difference between the MOSE-L and MOSE-L_TIC*ν*_ cells. A similar trend exists for the cell membrane conductance (${\bar{G}}_{mb}=\sigma_{mb}/t$), as it differs between the MOSE-E and MOSE-L and the MOSE-E and the MOSE-L_TIC*ν*_ cells, but not between the MOSE-L and the MOSE-L_TIC*ν*_ cells (Fig 4B). Additionally, the electrical conductivity of the cytoplasm slightly increases at the largest differences in malignant phenotype (from MOSE-E, to MOSE-L_TIC*ν*_), but not in the intermediate stages (between the MOSE-E and MOSE-L cells) (Fig 4C). However the large standard deviation indicate that this relationship may be less robust than the other relationships. Morphologically, the MOSE-E cells are also larger than the MOSE-L and MOSE-L_TIC*ν*_ cells, which are themselves similar in size.

#### Electrorotation for analysis of individual cells

**Fig 5:**
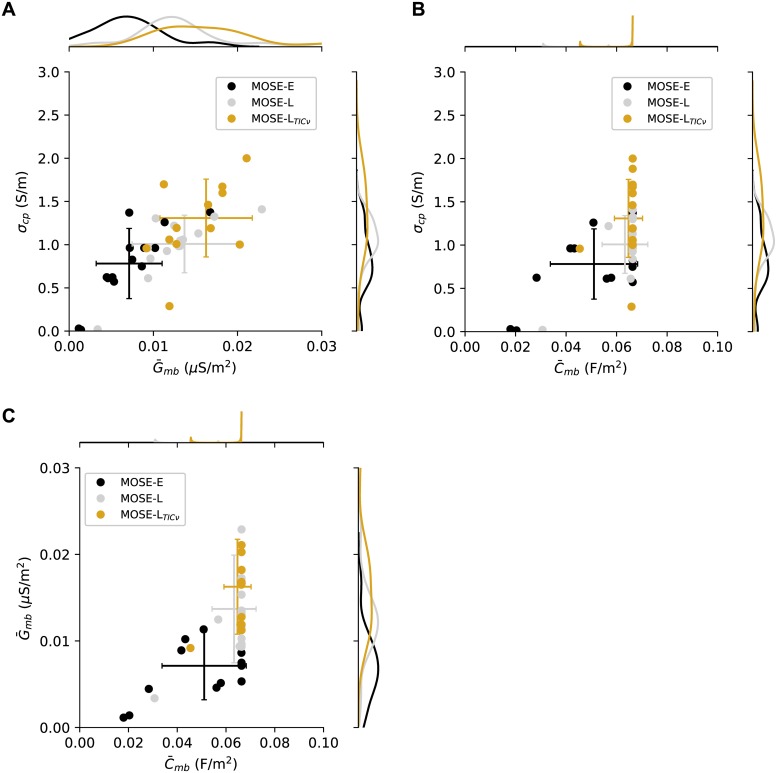
Shifts in cell electrical properties including membrane conductance, and cytoplasmic conductivity, and membrane capacitance are associated with the increasingly malignant phenotypes of the MOSE-E, MOSE-L, and MOSE-L_TIC*ν*_ cells. When the single-shell model was fit to experimental data from for cell individually, the paired kernel density estimates (KDE) of A: cytoplasmic conductivity (*σ*_*cp*_) and membrane conductance (${\bar{G}}_{mb}$), B: cytoplasmic conductivity and membrane capacitance (${\bar{C}}_{mb}$), and C: membrane conductance and membrane capacitance demonstrate shifts in the dielectric phenotype associated with increasingly malignancy. Error bars are centered at the mean for the cell type and represent the standard deviation. Calculated for each cell, the MOSE-E, *σ*_*cp*_ = 0.78±0.41 S/m, ${\bar{G}}_{mb}=0.71\pm 0.39$ S/cm^2^, and ${\bar{C}}_{mb}=0.051\pm 0.017$ F/m^2^. For MOSE-L, *σ*_*cp*_ = 1.01±0.33 S/m, ${\bar{G}}_{mb}=1.37\pm 0.62$ S/cm^2^, and ${\bar{C}}_{mb}=0.063\pm 0.009$ F/m^2^. For MOSE-L_TIC*ν*_, *σ*_*cp*_ = 1.31±0.45 S/m, ${\bar{G}}_{mb}=1.62\pm 0.55$ S/cm^2^, and ${\bar{C}}_{mb}=0.065\pm 0.056$ F/m^2^. The Levenberg-Marquardt algorithm was uses for fitting data from individual cells.

In order to investigate EROT as a marker-less technique for identifying the phenotypic malignancy of individual cells, the rotational spectrum experimentally obtained from each cell was fit using the single-shell model. In this manner, parameters that optimally characterized each cell using the single-shell model were calculated, demonstrating the feasibility of using EROT to manipulate individual cells to obtain parameters characterizing their dielectric phenotypes. Three parameters—the membrane conductance ${\bar{G}}_{mb}$, the cytoplasmic conductivity *σ*_*cp*_, and membrane capacitance ${\bar{C}}_{mb}$—exhibit consistent changes as the apparent malignancy of the cell line increases (Fig 5). Calculated for each cell, the MOSE-E, *σ*_*cp*_ = 0.78±0.41 S/m, ${\bar{G}}_{mb}=0.71\pm 0.39$ S/cm^2^, and ${\bar{C}}_{mb}=0.051\pm 0.017$ F/m^2^. For MOSE-L, *σ*_*cp*_ = 1.01±0.33 S/m, ${\bar{G}}_{mb}=1.37\pm 0.62$ S/cm^2^, and ${\bar{C}}_{mb}=0.063\pm 0.009$ F/m^2^. For MOSE-L_TIC*ν*_, *σ*_*cp*_ = 1.31±0.45 S/m, ${\bar{G}}_{mb}=1.62\pm 0.55$ S/cm^2^, and ${\bar{C}}_{mb}=0.065\pm 0.056$ F/m^2^. The MOSE-E cells have the lowest maximum in their membrane conductance kernel density function (KDE) and the MOSE-L_TIC*ν*_ have the highest maximum. The MOSE-L cells display a cytoplasmic conductivity KDE maximum between the MOSE-E and MOSE-L_TIC*ν*_ cells. These parameter shifts are consistent with their lineage of the MOSE-L cells as precursors to the MOSE-L_TIC*ν*_ and descendents of the MOSE-E cells.

While the purpose of the present study is to demonstrate the feasibility of discriminating between cells with different phenotypic malignancies, we characterized the grouping of cellular properties to identify candidates for use in future studies. When the membrane conductance, cytoplasmic conductivity, and cell radius are plotted as surfaces, clear shifts in these parameters are present in the transitions between the MOSE-E and the MOSE-L cells and the MOSE-L and the MOSE-L_TIC*ν*_ cells (Fig 5).

## Discussion

The mouse ovarian surface epithelial cells have been developed as an *in vitro* model of the pathogenesis of tumor cell malignancy. In particular, these cells have been shown to exhibit increasing cytoskeletal disorganization, altered serine and tyrosine phosphorylation, and expression and localization of signaling molecules [12]. Envisioning biological structures as an ensemble of charged species alludes to the presence of measurable bioelectrical signatures resulting from the physiological changes that occur in cells of populations associated with different malignancies. Previously, dielectrophoretic enrichment and separation of MOSE cells has demonstrated that these cells do indeed exhibit such bioelectrical signatures, specifically an increase in the specific capacitance of the cell membrane and an increase in the electrical conductivity of the cytoplasm [9, 27, 28]. Our results for the specific membrane capacitance are on the same order of magnitude as previous measurements [10, 27, 28] and confirms that these trends hold using EROT measurements of individual cells. Additionally, when each cell type was considered in aggregate (Table 2) and individually (Fig 5), the average cytoplasmic conductivity for the MOSE-E, MOSE-L, and MOSE-L_TIC*ν*_ cells reached similar values and showed a clear increase with increasingly malignant phenotype.

We allowed the conductivity, permittivity, and thickness of the cell membrane and the conductivity and permittivity of the cytoplasm to vary within a physiological range (Table 2), thereby introducing additional free parameters within our fitting algorithms. Our values for specific membrane capacitance and cytoplasmic conductivity were approximately twice the magnitude of the values previously reported in literature. These values are still within physiologically plausible range and have also taken into account direct measurements of the radius of each cell during fitting, rather than assuming a constant radius and using an average radius for each cell type for our analysis. Because an EROT spectrum was obtained for each cell individually, using both measurements of the applied frequency and the radius of each cell provides a robust parameter fit.

In addition to confirming that cytoplasmic conductivity and membrane specific capacitance increase with phenotypic malignancy, our analysis reveals that the specific conductance of the cell membrane also increases with malignancy, when each cell type was considered in aggregate (Table 2) and individually (Fig 5). Our data suggests that both the cell membrane permittivity and conductivity increase, though the membrane thickness remains largely constant or slightly decreases with increasingly malignancy, suggesting that the physical structure of the cell membrane becomes increasingly altered with an increasingly malignant phenotype (Table 1, Figs 4 and 5). In general, the difference between the MOSE-L and MOSE-L_TIC*ν*_ were more similar with the MOSE-L_TIC*ν*_ cells having a slightly greater membrane conductance and cytoplasmic conductivity, similar radius (Figs 4 and 5). However, the both the MOSE-L and MOSE-L_TIC*ν*_ appeared more different from the MOSE-E cells than from each other. The MOSE-E cells had an overall larger radius, lower cytoplasmic conductivity, lower membrane conductance, and lower membrane capacitance. As the MOSE-L and MOSE-L_TIC*ν*_ cells exhibit a more aggressive, malignant phenotype, than the early-stage MOSE-E cells, our results suggest that cell radius may decrease, cytoplasmic conductivity increases, and membrane conductance increases with an increasingly malignant phenotype. This finding could be particularly useful for developing high-throughput dielectrophoresis-based diagnostic screening [9] and in developing treatment planning algorithms for electroporation-based cancer therapies [49], which are specifically designed to isolate and destroy malignant cells.

These properties, specifically the membrane conductivity and permittivity describe not just the lipid bilayer, but also structures nearby that influence the electrical current, such as membrane proteins and cytoskeleton. Therefore, a change in cell rigidity may be expressed as a change in the membrane of a shell model. Interestingly, our data also indicates that variation in the cytoplasmic conductivity within a physiologically plausible range does not appear to influence the dielectric phenotype of the cell, as demonstrated by the large variation between the results of the two fitting algorithms (Table 1) and the agreement in the quality of fit of the single-shell model using the resulting parameter estimates (Table 2, Fig 2).

Electrorotation-based technologies inherently involve the cell-by-cell examination of an individual cell’s response to an applied AC electric field as its frequency is varied. The individualistic nature of this type of examination poses a particular challenge to scaling the technology for use in high-throughput screening or cellular enrichment applications. However, what EROT lacks in its ability to characterize a population of cells, it can be used to examine the properties of a single cell in high-fidelity. This cell-by-cell approach accounts for cell size in data fitting schemes, and eliminates it as an unspecified parameter in data fitting schemes. In DEP experiments cell size is often dominant and it is not possible to tease out other parameters.

The electrorotational spectra for an individual cell can provide a direct association between the electrophysiological properties of a cell and its physiology during a scan of a spectrum of applied electric field frequencies, while dielectrophoresis technologies are generally applied a single frequency or a summation of two or three frequencies [9, 50]. These data provide a larger information density for each cell examined and allows for stronger conclusions to be made about a cells behavior. In addition to a larger information density, the electrorotation device developed in this study is low-cost; it requires inexpensive instruments for waveform generation and relatively simple summation circuitry. Additionally, the electrode pattern can be fabricated using standard photolitography techniques, with feature sizes ≥ 50 *μ*m. The low-cost nature instrumentation, relatively simple fabrication, and the resolution of the EROT data position such electrotation-based devices to be valuable as a means of correlating electrophysiological measurements to the anatomical and physiological features that give rise to them.

## Conclusion

The microfluidic device developed for this work has been shown to successfully characterize the physiological properties of cancer cells at different stages of malignancy without the use of biochemical markers or additional pre-treatments. The method represents a fundamental technology for exploring the dielectric properties of individual cells; it is inexpensive to implement, requiring relatively large features for microfabrication techniques and simple instrumentation to trap and analyze cells based on their dielectric properties. The phenomenon of electrorotation in cells arises from and is intimately coupled to their morphology and electrophysiology. When tumor cells become more aggressive, their morphology is altered which changes the cell’s rotational velocity. Our data suggest that cell membrane conductance, capacitance, and cytoplasmic conductivity all increase with increasing phenotypic malignancy. These results indicate that dielectric spectroscopy may provide a means of characterizing the relationship between malignancy and electrophysiological changes during cancer progression.
